# Optimization of C-to-G base editors with sequence context preference predictable by machine learning methods

**DOI:** 10.1038/s41467-021-25217-y

**Published:** 2021-08-12

**Authors:** Tanglong Yuan, Nana Yan, Tianyi Fei, Jitan Zheng, Juan Meng, Nana Li, Jing Liu, Haihang Zhang, Long Xie, Wenqin Ying, Di Li, Lei Shi, Yongsen Sun, Yongyao Li, Yixue Li, Yidi Sun, Erwei Zuo

**Affiliations:** 1 Shenzhen Branch, Guangdong Laboratory for Lingnan Modern Agriculture, Genome Analysis Laboratory of the Ministry of Agriculture, Agricultural Genomics Institute at Shenzhen, Chinese Academy of Agricultural Sciences, Shenzhen, China; 2 Institute of Neuroscience, CAS Center for Excellence in Brain Science and Intelligence Technology, Chinese Academy of Sciences, Shanghai, China; 3 Department of Neurology and Institute of Neurology, First Affiliated Hospital, Institute of Neuroscience, Fujian Medical University, Fuzhou, China; 4 State Key Lab for Conservation and Utilization of Subtropical Agric-Biological Resources, Guangxi University, Nanning, China; 5 Bio-Med Big Data Center, Key Laboratory of Computational Biology, CAS-MPG Partner Institute for Computational Biology, Shanghai Institute of Nutrition and Health, Shanghai Institutes for Biological Sciences, University of Chinese Academy of Sciences, Chinese Academy of Sciences Shanghai, Shanghai, China

**Keywords:** Biotechnology, CRISPR-Cas systems

## Abstract

Efficient and precise base editors (BEs) for C-to-G transversion are highly desirable. However, the sequence context affecting editing outcome largely remains unclear. Here we report engineered C-to-G BEs of high efficiency and fidelity, with the sequence context predictable via machine-learning methods. By changing the species origin and relative position of uracil-DNA glycosylase and deaminase, together with codon optimization, we obtain optimized C-to-G BEs (OPTI-CGBEs) for efficient C-to-G transversion. The motif preference of OPTI-CGBEs for editing 100 endogenous sites is determined in HEK293T cells. Using a sgRNA library comprising 41,388 sequences, we develop a deep-learning model that accurately predicts the OPTI-CGBE editing outcome for targeted sites with specific sequence context. These OPTI-CGBEs are further shown to be capable of efficient base editing in mouse embryos for generating *Tyr*-edited offspring. Thus, these engineered CGBEs are useful for efficient and precise base editing, with outcome predictable based on sequence context of targeted sites.

## Abstract (web summary)

C->G transversions can be highly desirable editing outcomes. Here the authors optimise CGBEs and provide a deep learning model for predicting editing outcomes based on sequence context.

## Introduction

Precise alteration of single nucleotides is a powerful approach in gene editing for biological research and therapeutic applications^1^. Cytosine base editors (CBEs)^2^ and adenine base editors (ABEs)^3^ have been developed to enable C-to-T or A-to-G conversion at target sites, respectively. However, these BEs are unable to install C-to-G or A-to-T transversion, which may correct 40% of human pathogenic point mutations^4^. Two recent reports have shown C-to-G transversion could be achieved by replacing the uracil-DNA glycosylase inhibitor (UGI) of a CBE with an uracil-DNA glycosylase (UNG)^5,6^. While, these C-to-G editors showed efficient editing at limited target sites and provided few rules for efficient C-to-G editing.

In this study, we aim to further elevate C-to-G transversion efficiency by optimizing the design of CBEs. Starting with changing the species origin and relative position of uracil-DNA glycosylase and deaminase, we obtain OPTI-CGBEs for efficient C-to-G transversion. We determine the motif preferences of these OPTI-CGBEs using a sgRNA library comprising 41,388 sequences, and then develop a deep-learning model that accurately predicts the OPTI-CGBE editing outcome for targeted sites with specific sequence context. Finally, we demonstrate the capability of these OPTI-CGBEs for efficient base editing in mouse embryos. These CGBE variants expand the scope of base editing and provide selection criteria for future gene editing that requires C-to-G transversion.

## Results

### Generation of CGBE variants by rational gene engineering

**Fig. 1:**
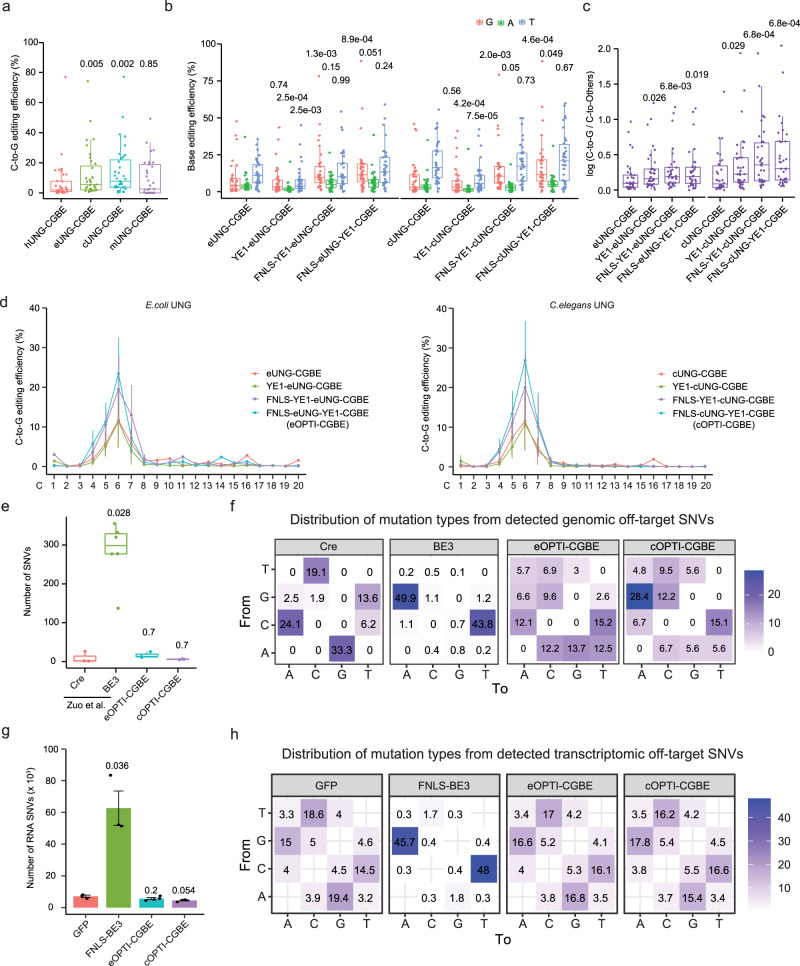
Engineering of CGBEs. **a** The C-to-G transversion efficiency of engineered CGBEs with different UNGs at 34 endogenous target sites in HEK293T cells. hUNG for human UNG, eUNG for *E.coli* UNG, cUNG for *C.elegans* UNG, mUNG for mouse UNG. *P* values above each group indicated the comparison with hUNG-CGBE group. **b** The base editing efficiency of engineered CGBEs at 34 endogenous target sites in HEK293T cells. YE1 = W90Y + R126E. *P* values above each group indicated the comparison with eUNG-CGBE or cUNG-CGBE group. **c** The log transformed ratios of C-to-G/C-to-Others editing among engineered CGBEs. *P* values above each group indicated the comparison with eUNG-CGBE or cUNG-CGBE group. The center line indicates the median, and the bottom and top lines of the box represent the first quartile and third quartile of the values, respectively. Tails extend to the minimum and maximum values. **d** The C-to-G transversion efficiency of engineered CGBEs at each protospacer position 1–20 (where PAM is at positions 21–23) of 34 endogenous target sites. *n* = 3 biological replicates for each site. Data are presented as mean values ± SEM. **e** Comparison of the total number of detected SNVs on DNA level. *n* = 3 for Cre, eOPTI-CGBE and cOPTI-CGBE group, and *n* = 6 for BE3 group. *P* values above each group indicated the comparison with Cre group. The center line indicates the median, and the bottom and top lines of the box represent the first quartile and third quartile of the values, respectively. Tails extend to the minimum and maximum values. **f** Distribution of mutation types from detected SNVs for indicated groups. **g** Comparison of the total number of detected RNA SNVs among different groups. *n* = 3 for GFP and FNLS-BE3 groups, *n* = 4 for eOPTI-CGBE and cOPTI-CGBE groups. Data are presented as mean values ± SEM. *P* values above each group indicated the comparison with GFP group. **h** Distribution of mutation types from detected RNA SNVs for groups transfected with GFP, FNLS-BE3, eOPTI-CGBE, or cOPTI-CGBE plasmid. All *P* values were calculated by two-sided Wilcoxon rank sum tests.

We first compared the efficiency of C-to-G base editing using UNGs from human, *E. coli*, mouse, or *C. elegans* to substitute UGI of BE3 (Supplementary Fig. 1). For 34 endogenous sites in HEK293T cells, we found that C-to-G BE (CGBE) variants with the *E. coli* or *C. elegans* UNG (eUNG or cUNG) achieved much higher C-to-G transversion efficiency than that with human UNG (Fig. 1a).

Previous reports have shown that BE3 induced a substantial amount of random DNA and RNA point mutations^7–10^, and the extent of such off-target effects can be reduced by introducing mutations into the ssDNA binding domain of BE3’s deaminase rAPOBEC1^11,12^. Thus, we introduced mutations W90Y and R126E into the rAPOBEC1 module of CGBEs (abbreviated YE1)^11,12^ to generate two variants: YE1-eUNG-CGBE, and YE1-cUNG-CGBE (Supplementary Fig. 1). Testing of editing efficiency for the 34 target sites in HEK293T cells showed that the bystander C-to-A and C-to-T edits of the two variants were substantially reduced compared to the original CGBEs with the wild-type rAPOBEC1 (Fig. 1b). Besides, the purity of editing products (C-to-G divided by C-to-others editing efficiency) was significantly increased in the two CGBE variants (Fig. 1c).

To further improve the editing efficiency of YE1-eUNG-CGBE or YE1-cUNG-CGBE, we modified the proteins by adding a nuclear location signal peptide and optimizing the codons for expression in human cells^13^. The higher expression level of the variant (FNLS-YE1-eUNG-CGBE) resulted in higher overall editing efficiency in HEK293T cells compared with YE1-eUNG-CGBE (two-fold; Fig. 1b). The further change in domain position by fusing the eUNG to the N-terminus of CGBE (FNLS-eUNG-YE1-CGBE) instead of the original C-terminal location resulted in further elevation of editing efficiency (to 22.7% on average; Fig. 1b). Similarly, an improved version of FNLS-cUNG-YE1-CGBE carrying cUNG at the N-terminus also significantly improved the C-to-G editing efficiency of YE1-cUNG-CGBE (3-fold; Fig. 1b). The products purity was also significantly increased for both FNLS-eUNG-YE1-CGBE and FNLS-cUNG-YE1-CGBE (Fig. 1c). Moreover, we found FNLS-eUNG-YE1-CGBE and FNLS-cUNG-YE1-CGBE showed a narrowed editing window that spanned protospacer positions 4–7, with protospacer adjacent motif (PAM) spans positions 21–23 (Fig. 1d), a feature preferable for base editing^14^. We next compared our constructs with the CGBE construct (eUNG-BE4max(R33A)∆UGI; referred as CGBE1) from Kurt et al.^6^ and found that our optimized CGBEs showed significantly higher C-to-G editing efficiency on the tested target sites (Supplementary Fig. 2d). Specifically, our optimized CGBEs showed higher editing efficiencies at positions 5 and 6 within the editing window (Supplementary Fig. 2e). Moreover, both FNLS-eUNG-YE1-CGBE and FNLS-cUNG-YE1-CGBE produced editing products with higher purity, as the ratio between C-to-G and C-to-others edits were significantly increased compared with CGBE1 (Supplementary Fig. 2f). The indel frequency of FNLS-cUNG-YE1-CGBE was significantly reduced simultaneously (Supplementary Fig. 2g). Besides, we compared our CGBEs with prime editors (PE2 and PE3) that can introduce a diverse range of different edits^15,16^. Across six different target sites that we tested in this comparison experiment, we found that both PE2 and PE3 were substantially less efficient than our optimized CGBEs (Supplementary Fig. 2h), and PE3 also induced higher frequencies of indel edits (Supplementary Fig. 2i).

We next applied GOTI^7^ and RNA-seq methods^9^ to assess the potential DNA and RNA off-target effects of FNLS-eUNG-YE1-CGBE and FNLS-cUNG-YE1-CGBE. The numbers of SNVs in CGBE-edited mouse embryos were similar to those at the spontaneous SNV level, and is much lower than that of BE3 group, which was known to induce off-target SNVs^11^ (Fig. 1e). Besides, no mutation bias was observed in the CGBE groups (Fig. 1f), indicating that our engineered CGBE variants induced no detectable off-target effects on DNA level. On the other hand, cells treated with FNLS-eUNG-YE1-CGBE or FNLS-cUNG-YE1-CGBE showed no increased number of RNA SNVs and no mutation bias compared with control cells (Fig. 1g, h), suggesting that the engineered CGBE variants induced no RNA off-target effects. In addition, our target sequencing data also revealed no obvious sgRNA-dependent off-targets predicted from Cas-OFFinder^17^ (Supplementary Fig. 3). Together, these results revealed that FNLS-eUNG-YE1-CGBE and FNLS-cUNG-YE1-CGBE, termed hereafter as eOPTI-CGBE and cOPTI-CGBE, respectively, could achieve high C-to-G transversion efficiency with low off-target effects.

### Motif preference analysis of OPTI-CGBEs

**Fig. 2:**
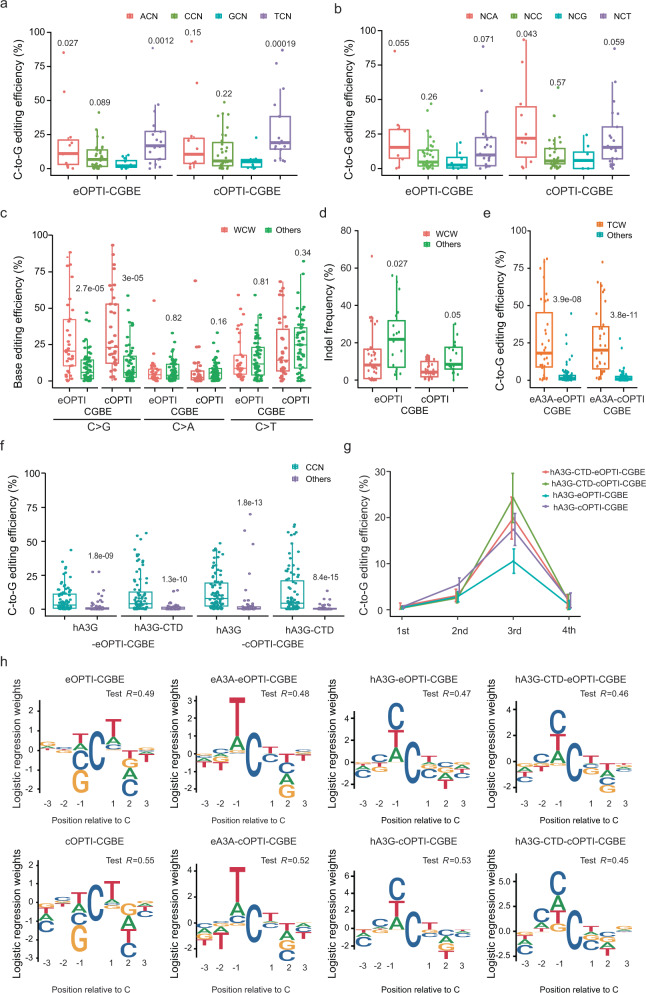
Motif analysis of OPTI-CGBEs. **a** The C-to-G transversion efficiency induced by eOPTI-CGBE or cOPTI-CGBE of targeted Cs bearing different nucleotides 1nt upstream. N = A, T, G, or C. *P* values above each group were calculated between the group with “GCN” group. **b** The C-to-G transversion efficiency induced by eOPTI-CGBE or cOPTI-CGBE of targeted Cs bearing different nucleotides 1nt downstream. *P* values above each group were calculated between the group with “NCG” group. **c** Comparison of base editing efficiency of eOPTI-CGBE or cOPTI-CGBE at “WCW” or other motif of the 34 original and 20 additional target sites. W = A or T. **d** Indel frequency of eOPTI-CGBE or cOPTI-CGBE at “WCW” or other motif of the 34 original and 20 additional target sites. **e** Comparison of C-to-G editing efficiency of OPTI-CGBEs with eA3A deaminase at “TCW” or other motif of the 34 original and 20 additional target sites. **f** Comparison of C-to-G editing efficiency of OPTI-CGBEs with hA3G or hA3G-CTD deaminase at “CCN” or other motif of the 34 original target sites and 26 additional target sites. The center line indicates the median, and the bottom and top lines of the box represent the first quartile and third quartile of the values, respectively. Tails extend to the minimum and maximum values. *n* = 3 biological replicates for each site. All *P* values were calculated by two-sided Wilcoxon rank sum tests. **g** C-to-G editing efficiency of each C induced by OPTI-CGBEs with hA3G or hA3G-CTD deaminase when the target sites had more than 2 Cs. *n* = 3 biological replicates for each site. Data are presented as mean values ± SEM. **h** Motif logo detected by a logistic regression model developed with a training dataset (80%) sampled from the detected base editing activities with the paired sgRNA library. The *y*-axis represents learned weights from the regression model for each nucleotide.

So far, we were simply assessing the editing success rate across the 34 target sites. However, when we specifically examined the sequence context information of the successfully eOPTI-CGBE-edited sites, we detected an obvious preferential 3nt motif (“WCW”; W could be either A or T) conversion with no increase of bystander edits (Fig. 2a, b and Supplementary Fig. 4a, b). A very similar preferential motif was detected for cOPTI-CGBE, albeit with a slightly more pronounced preference for T over A in the W position (Fig. 2a and Supplementary Fig. 4a, b).

We next conducted editing experiments in which we targeted 20 additional target sites that all contained this preferential “WCW” motif. In support of this notion, we found significantly higher on-target eOPTI-CGBE editing efficiency for targeted Cs with the motif, as compared to that without the motif (3.2 fold; Fig. 2c). A similar elevation in on-target editing efficiency was also detected for cOPTI-CGBE editing (2.8 fold; Fig. 2c). Interestingly, this comparative analysis of the “WCW”-motif-bearing target sites also revealed a substantial reduction in the frequency of bystander edits and induced indels for both eOPTI-CGBE and cOPTI-CGBE (Fig. 2c, d). Thus, eOPTI-CGBE and cOPTI-CGBE can achieve very high on-target C-to-G editing efficiency when targeting sites bearing “WCW” motifs, with high product purity.

Due to the relatively limited scope of target sitesx examined above, we further examined the motif-dependent editing for other deaminases in order to expand the targeting scope of C-to-G editors using targeted C within 3nt motifs other than WCW. We explored three different deaminase modules (Supplementary Figs. 4c and 5a): a mutated human APOBEC3A which showed “TCN” motif preference^18^ and two variants of APOBEC3G module (hA3G-OPTI-CGBE and hA3G-CTD-OPTI-CGBE), which preferred C-enriched sequences^19,20^. We firstly analyzed the editing efficiency and activity window of these CGBE editors at the 34 target sites examined in the above experiments (Supplementary Figs. 4d–g and 5b–f). Not surprisingly, these engineered CGBEs also showed a narrowed editing window similar to that of eOPTI-CGBE and cOPTI-CGBE (Supplementary Figs. 4d and 5b). Besides, both eA3A-eOPTI-CGBE and eA3A-cOPTI-CGBE showed an obvious preference to “TCW”, where W was A or T (Fig. 2e and Supplementary Fig. 4e, f), in line with the motif preference of cOPTI-CGBE.

In contrast to the motif preference of APOBEC1 and APOBEC3A, we found that the two variants of APOBEC3G constructed with *E. Coli* UNG (hA3G-eOPTI-CGBE and hA3G-CTD-eOPTI-CGBE) had significant preferences for the “CCN” motif, where N could be any nt, as shown by markedly (~3–5 fold) higher editing efficiency for target sites bearing the CCN motif, in comparison with those without this motif (Fig. 2f and Supplementary Fig. 5c–e). Similar results were observed for hA3G-cOPTI-CGBE and hA3G-CTD-cOPTI-CGBE, constructed with UNG from *C. elegans* (Fig. 2f and Supplementary Fig. 5c–e). In addition, when the target site comprised three or more consecutive Cs, the efficiency of C-to-G conversion was the highest for the third C, for APOBEC3G variants containing either eUNG or cUNG (Fig. 2g and Supplementary Fig. 5f). This was not the case for C-to-T editing by hA3G-CBE, which is known to prefer the conversion of second C^20^.

To further broaden the targeting scope of C-to-G editing, we also constructed 6 CGBE-NG editors, which could identify “NG” PAM by replacing Cas9n with Cas9n-NG^21^, spGn^22^, or xCas9n^23^ (Supplementary Fig. 6a). We found that the editing efficiency of Cas9n-NG and spGn were higher than the xCas9n version (Supplementary Fig. 6b, d), and the indel frequency was lower in Cas9n-NG than that in spGn (Supplementary Fig. 6c). Cas9n-NG is thus the best version for C-to-G editing at sites with NG PAMs.

### Editing outcome prediction of OPTI-CGBEs by computational methods

We performed a large-scale screen to assess the motif preference of eOPTI-CGBE and cOPTI-CGBE, using a previously developed paired sgRNA library of 41,388 cloned oligonucleotides, each comprising a 20nt sgRNA sequence together with its targeting sequence^24^. HEK293T cells were infected with lentiviral vectors containing the paired sgRNA library, followed by transfection with a plasmid encoding one of the eight OPTI-CGBEs. Deep sequencing was then performed to assess editing outcomes and to explore impact of the sequence context on editing efficiency. For sites with targeted Cs spanning 4–7 positions of the protospacer and more than 100× coverage, we found that the motif preference for the library sequences were largely consistent with that found for the endogenous sites examined above (Supplementary Fig. 7a–d): eOPTI-CGBE and cOPTI-CGBE preferred “WCW” motif (Supplementary Fig. 7a), eA3A-OPTI-CGBEs preferred “TCW” motif sites (Supplementary Fig. 7b), and APOBEC3G variants preferred “CCN” motif (Supplementary Fig. 7c, d).

We next built a logistic regression model to learn the motif preference using a training dataset (80% randomly sampled) from the paired sgRNA library. The model was then tested with the rest 20% of the library and showed good performance. The learned parameters are visualized by sequence logos, showing the motif preferences of eOPTI-CGBE (for WCW), cOPTI-CGBE (for TCW), eA3A-OPTI-CGBEs (for TCW), and hA3G-OPTI-CGBEs (for CCN) (Fig. 2h). These results indicated the impact of sequence context on the editing efficiency of OPTI-CGBEs, for 20–30% of the variance in editing efficiency could be explained by target motifs in the test dataset (variance explained = *R*^2^; Fig. 2h).

**Fig. 3:**
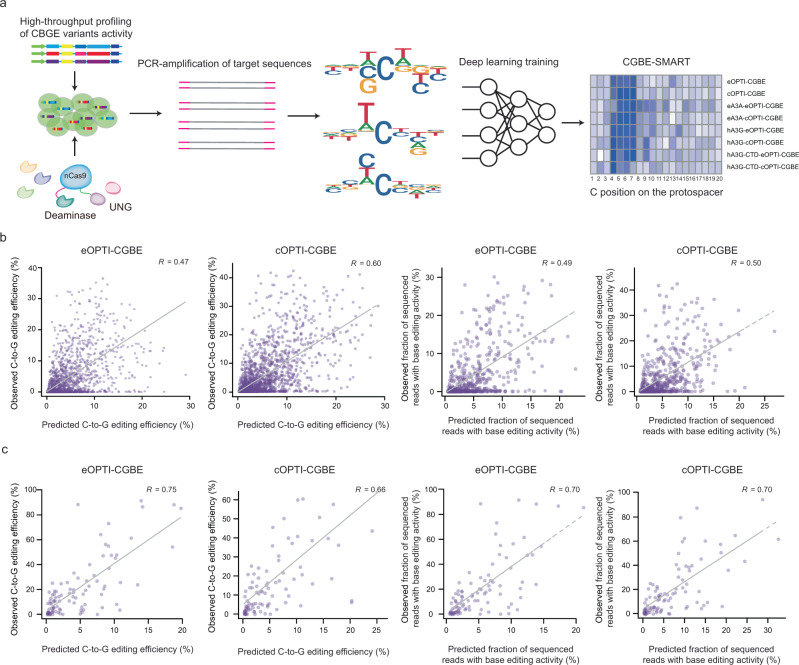
Machine-learning models of OPTI-CGBEs. **a** Model design for predicting C-to-G base editing efficiency. **b** Comparison of predicted versus observed base editing efficiency or faction of sequenced reads with base editing activities at target sites of OPTI-CGBEs using lentiviral paired sgRNA library. **c** Comparison of predicted versus observed base editing efficiency or faction of sequenced reads with base editing activities at 80 endogenous target sites of OPTI-CGBEs. *R* values demonstrated Pearson’s correlation coefficients.

In order to determine the best base editor for targeting novel sequences, it would be of great value to develop a computing algorithm that predicts the editing efficiency of CGBEs based on the sequence context of targeted sites. Deep-learning methods have been successfully used to predict the editing outcome for spCas9^25^, C-to-T and A-to-G base editors^26,27^. We thus designed and trained a deep neural network for C-to-G base editors. The neural network model, termed “CGBE-SMART” (http://www.sunlab.fun:3838/BE_SMART/), accepts an input target sequence surrounding a protospacer and PAM and outputs both the per-site C-to-G editing efficiency and the probability of each editing outcome (Fig. 3a and Supplementary Fig. 8). For each position in the target site, we designed networks with window sizes from 7 to 11 so that the model could focus more on the impact of adjacent nucleotides. The final output was the weighted average of the results from these networks. The efficiency model (CGBE-SMART_Efficiency) was trained by minimizing the mean square error (MSE) between observed C-to-G editing efficiency and predicted values (Supplementary Fig. 8). Then we applied a bayesian network to infer the dependency between each two edited positions and further output the proportion of all outcomes (CGBE-SMART_Proportion; Supplementary Fig. 9). We split the dataset into a training set, a validation set and a testing set by proportion of 6:1:3, and a separate model was trained for each of the eight OPTI-CGBEs. The performance of each model was evaluated on the independent test datasets using pearson’s correlation coefficients between predicted and observed C-to-G editing efficiency at each targeted C or proportions of editing outcomes (Fig. 3 and Supplementary Fig. 9). Consistent with our findings earlier, we found that higher prediction accuracy was observed for Cs within the target window of 4–7nt than those beyond (Supplementary Fig. 9a). Generally, we found that CGBE-SMART achieved high prediction accuracy on editing outcomes of the target sequences in the test dataset (*R* = 0.20–0.60 for CGBE-SMART_Efficiency; *R* = 0.37–0.60 for CGBE-SMART_Proportion; Fig. 3b and Supplementary Fig. 9b, c). Among the eight OPTI-CGBEs, cOPTI-CGBE showed the best performance between predicted editing efficiencies and observed ones in CGBE-SMART_Efficiency model (Fig. 3b). Simultaneously, eA3A-cOPTI-CGBE achieved a correlation coefficient of 0.6 between the predicted proportions of editing outcomes and observed ones in CGBE-SMART_Proportion model (Supplementary Fig. 9c). In comparison with the deep conditional autoregressive model from BE-Hive^27^ or DeepCBE^26^, we found that CGBE-SMART showed much higher prediction accuracy for seven CGBE editors except for hA3G-CTD-cOPTI-CGBE model (averaged *R* = 0.47 vs. 0.15 vs. 0.33; Supplementary Fig. 9d). Since CGBE-SMART can be trained on different observed data and predict editing outcome for novel inputs, we next applied our model to predict C-to-T editing efficiency and compared with BE-Hive and DeepCBE using the test datasets from the two corresponding studies^26,27^ (see Methods). We found that CGBE-SMART achieved high prediction accuracy in C-to-T editing efficiency for all the four datasets (averaged *R* = 0.75; Supplementary Fig. 9e). In comparison with BE-Hive, CGBE-SMART achieved much higher prediction accuracy for BE4-CP dataset, similar performance for BE4 dataset, but lower prediction accuracy for BE4max dataset (Supplementary Fig. 9e). CGBE-SMART achieved high performance in C-to-T base editing efficiency prediction for HT_CBE_Test dataset similar to DeepCBE (*R* = 0.69 vs 0.67; Supplementary Fig. 9e). These results represent that CGBE-SMART is a general method for modeling and comparing the on-target accuracy of different base editors.

The evaluation of CGBE-SMART above is only based on artificial sequence library, so we further tested the generalization of the model using natural genomic targets as inputs. We applied the trained CGBE-SMART model to predict the C-to-G editing efficiency of the 80 endogenous sites examined in above experiments, and found a high correlation (averaged *R* = 0.64) between predicted and experimentally observed editing efficiencies (Fig. 3c and Supplementary Fig. 9f). Similarly, CGBE-SMART_Proportion also achieved great performance on predicting the fraction of sequenced reads with base editing frequency for the eight CGBEs (averaged *R* = 0.66; Fig. 3c and Supplementary Fig. 9g). Taken together, we have shown that our CGBE-SMART model is capable of predicting editing efficiencies and outcomes for both exogenous and endogenous target sites. Although base editing efficiency is subjected to various experimental conditions such as the cell type and transfection efficiency, the editing of exogenous and endogenous target sites follows similar patterns.

### C-to-G editing of OPTI-CGBEs in mouse embryos

**Fig. 4:**
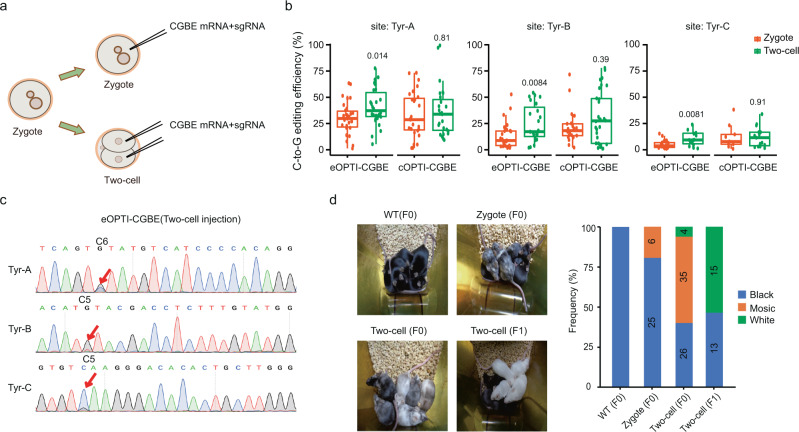
Application of OPTI-CGBEs for C-to-G editing in mice embryos. **a** Schematics of zygote and two-cell injection of OPTI-CGBEs. **b** The C-to-G transversion efficiency of OPTI-CGBEs on three target sites by zygote or two-cell injections. *n* = 30/30/27/27 for Tyr-A, *n* = 28/23/30/21 for Tyr-B, and *n* = 21/15/9/15 for Tyr-C, respectively (Ordered: eOPTI-CGBE-Zygote, eOPTI-CGBE-Two-cell, cOPTI-CGBE-Zygote, and cOPTI-CGBE-Two-cell). The center line indicates the median, and the bottom and top lines of the box represent the first quartile and third quartile of the values, respectively. Tails extend to the minimum and maximum values. *P* values were calculated by two-sided Wilcoxon rank sum tests. **c** Sanger sequencing results of eOPTI-CGBE on three target sites by two-cell injection. Red arrows indicated the targeted Cs. **d** The hair colors of F0 and F1 mice by zygote or two-cell injection of eOPTI-CGBE mRNA and sgRNA-Tyr-C. The numbers on each bar represent the number of mice. *P* value was calculated by Chi-square test.

Having obtained CGBE variants that exhibited high editing efficiency for C-to-G base editing under various sequence contexts and very few off-target effects, we then used these variants to edit genomic DNA in mouse embryos. The mRNA encoding eOPTI-CGBE or cOPTI-CGBE was injected into zygotes, together with one of three selected sgRNAs (Fig. 4a). The embryonic development was not deleteriously affected by the injection (Supplementary Fig. 10a). We first found that both CGBE variants achieved high C-to-G base transversion efficiency for three targeted sites on Tyr gene (Fig. 4b). In light of previous reports on optimizating gene editing in mice^28^, we tested OPTI-CGBE-mediated base editing in two-cell stage embryos. Consistent with previous findings, the C-to-G transversion efficiency of OPTI-CGBEs was indeed substantially increased by injection at the two-cell stage for all the three Tyr target sites (Fig. 4b), as further validated by Sanger sequencing (Fig. 4c). Notably, we found that the indel frequency of cOPTI-CGBE was much lower than that of eOPTI-CGBE (Supplementary Fig. 10c), consistent with our observation in HEK293T cells (Fig. 2d). We also applied CGBE-SMART to predict the C-to-G editing efficiency on the three target sites based on the sequence content, and found good agreement of two of the three Tyr sites (Tyr-A and Tyr-B). The predicted efficiency for Tyr-C was much higher than the observed one (Supplementary Fig. 10b), presumably resulting other in vivo factors other than the sequence context.

We have also examined the phenotypic consequence of the higher editing efficiency of OPTI-CGBEs in *Tyr* gene-edited mice. Tyr-C editing introduced a stop codon on *Tyr* gene that results in the Albino phenotype in C57BL/6J mice^29^. We injected eOPTI-CGBE mRNA and sgRNA-Tyr-C in either zygotes or two-cell embryos, transplanted the embryos into recipient mothers, and tracked the hair-color phenotype of pups. Consistent with the earlier editing efficiency analysis, we found that pups derived from two-cell-injected embryos also showed higher C-to-G editing rates at the *Tyr* gene, as compared to those from zygote-injected embryos (Fig. 4d). Furthermore, pups derived from zygote-injection embryos mostly wild-type (WT) black hair and a small percentage of black-white mosaic hair, whereas those derived from two-cell-injected embryos mostly showed much larger fraction of mosaic hair and small percentage of uniformly white hair (Fig. 4d). Mating mosaic hair females and males from the latter group produced more than 50% of white-hair offspring, and no offspring with mosaic hair (Fig. 4d). These results demonstrate that OPTI-CGBEs is an efficient tool for genome base editing in mammalian embryos.

## Discussion

By using the UNG domain from species and shuffling sequences and positions of deaminase domain in various base editors, we obtained engineered CGBE variants (OPTI-CGBEs) that achieve both high C-to-G transversion efficiency and low off-target effects. Our OPTI-CGBEs outperformed previously reported CGBE1^6^ and prime editors^15,16^ in C-to-G editing efficiency and product purity across the tested target sites. Very recently, Chen et al. reported a C-to-G base editor by replacing the UGI of BE3 with base excision repair (BER) proteins with improved C-to-G editing efficiency at specific motifs^30^. Notably, in our study we found that OPTI-CGBEs differ from corresponding CBEs in their motif preferences, and CGBEs with deaminases of different origins prefer distinct sequence context. The motif preferences of these C-to-G base editors would possibly be explained by the distinct binding modes adopted by the corresponding deaminases of different CGBEs. The increased C-to-U editing by deaminases thus increased the C-to-G editing efficiency of CGBEs. We also conducted high-throughput analysis of the editing efficiency of these variants using a DNA library containing 41,388 target sequences, in order to elucidate their motif preferences. Two computational methods, including a deep-learning model (CGBE-SMART), were developed for predicting C-to-G editing efficiency and proportions of editing outcomes. The CGBE-SMART model enabled effective sgRNA selection at target sites with specific sequence context, and could be generalized to support efficient sgRNA selection for optimal use of BEs. Indeed, CGBE-SMART also achieved high performance in predicting editing efficiency for CBEs, comparable to previously developed deep-learning models for the same purposes^26,27^. While, CGBE-SMART showed better performance in our CGBE datasets than other models (Fig. 3 and Supplementary Fig. 9), which could probably be explained by that CGBE-SMART was designed for predicting C-to-G editing efficiencies and have taken the characteristics of CGBEs into consideration. This empirical discovery of the sequence motif preference of BEs points to an important aspect in engineering BEs with optimal base editing. Nevertheless, the low C-to-G editing efficiencies in the high-throughput analyses would underrate the performance of computational model. Our studies in HEK293T cells and in embryos also suggest that the editing outcome could be affected by in vivo factors like epigenetic regulation, chromatin accessibility and DNA repair activities, which deserve to be further examined in addition to sequence context of the target site. In this work, we demonstrated the high C-to-G transversion efficiency for diverse sequence context and minimal off-target effects of a group of optimized CGBE variants, and their efficiency in producing genome-edited offspring. Guided by the computational algorithm we have developed for predicting editing efficiency based on sequence motif, these CGBE variants may prove to be valuable for future gene editing that requires C-to-G transversion.

## Methods

### Animals

Four-week-old female mice were maintained in a SPF facility under a 12 h dark-light cycle and mated with male mice. Female mice were used for embryo collection. The animal usage and care complied with the guideline of the Biomedical Research Ethics Committee of Shanghai Institutes for Biological Science, Chinese Academy of Sciences.

### Plasmid construction and cloning

pCMV-BE3 (Addgene plasmid#73021) and pCMV-YE1-FNLS-BE3 (Addgene Plasmid #154005) were used as backbones. A CMV-mCherry expression cassette was inserted into backbone plasmid and the sequence encoding UGI was replaced by codon optimized UNG sequence (Genewiz). Site-directed mutagenesis was performed using NEBuilder HiFi DNA Assembly Master Mix (New England BioLabs) for constructing plasmids expressing different CGBEs. U6-sgRNA-scaffold-pCMV-EGFP-poly A was generated through NEBuilder HiFi DNA Assembly, by combining a PCR-amplified U6-sgRNA-scaffold with a digested pCMV-EGFP-poly A backbone. The amino-acid sequence for OPTI-CGBEs was supplied in Supplementary Data 1.

### Cell culture, transfection, and FACS

HEK293T (ATCC#: CRL-3216) cells were cultured in Dulbecco’s modified Eagle medium (DMEM, Gibco) supplemented with 10% FBS (BI) and 1% penicillin/streptomycin (Gibco) at 37 °C in 5% CO_2_ incubators. The pCMV-CGBE variants-poly A-pCMV-mCherry-poly A and U6-sgRNA-scaffold-pCMV-EGFP-poly A plasmids were co-transfected using polyethyleneimine (PEI, Polyscience) according to the manufacturer’s protocols. Forty-eight hours after transfection, cells were washed with PBS and digested with 0.25% trypsin (Gibco). Then cells were filtered with a 40 μm cell strainer. The mCherry and GFP double-positive cells were sorted by flow cytometer (FlowJo X 10.0.7). The gating strategy in the identification of GFP^+^ and mCherry^+^ cells for on-target editing efficiency evaluation was supplied in Supplementary Fig. 2a.

### Lentivirus production and transduction

Paired sgRNA library was a gift from Dr. Leopold Parts in Wellcome Sanger Institute. For lentivirus production, supernatants containing lentiviral particles were collected 48 h after transfecting HEK293T with 30 μg paired sgRNA lentiviral vector, 22.5 μg psPAX2 and 15 μg pMD2.G in a 15 cm dish. For lentiviral transduction of HEK293T cells, paired sgRNA library cell lines were incubated with the lentiviral supernatant. HEK293T cell line stably expressing the paired sgRNA library (HEK293T-sgRNA-library) was generated by lentiviral transduction at MOI 0.3 followed by selection in the presence of 2 μg/ml puromycin. The HEK293T-sgRNA-library cell lines were next transfected with base editor plasmids expressing mCherry, and positive cells were collected by FACS according to the expression level of mCherry. Genomic DNA was next extracted from the mCherry^+^ cells using TIANamp Genomic DNA Kit (TIANGEN) according to the manufacturer’s protocols. Sites of interest were amplified by nested PCR using gene-specific primers (Supplementary Table 1) flanking the target sequence. PCR products were purified using universal DNA purification kit (TIANGEN) according to the manufacturer’s instructions. The PCR products were then ligated to adapters and sequencing was performed on the Illumina HiSeq X Ten platform.

### In vitro transcription of OPTI-CGBE mRNA and sgRNA

T7 promoter was added to the coding region of OPTI-CGBE by PCR amplification from plasmid expressing OPTI-CGBE, using CGBE-F and CGBE-R primer. T7-OPTI-CGBE PCR product was purified and used as the template for in vitro transcription (IVT) using mMESSAGE mMACHINE T7 ULTRA kit (Life Technologies). T7 promoter was added to sgRNA template by PCR amplification of px330 using primer Tyr-IVT-F and sgRNA IVT-R. The T7-sgRNA PCR product was purified and used as the template for IVT using MEGA shortscript T7 kit (Life Technologies). OPTI-CGBE mRNA and sgRNAs were purified using MEGA clear kit (Life Technologies), eluted in RNase-free water and stored at −80 °C.

**Table:** 

CGBE-F: 5’-TCCGCGGCCGCTAATACGACT-3’
CGBE-R: 5’-TGGTTCTTTCCGCCTCAGAAGCC-3’
Tyr-A-IVT-F:
5’-TAATACGACTCACTATAGGGTCAGTCTATGTCATCCCCACGTTTTAGAGCTAGAAATAG-3’
Tyr-B-IVT-F: 5’-TAATACGACTCACTATAGGGACATCTACGACCTCTTTGTAGTTTTAGAGC
TAGAAATAG-3’
Tyr-C-IVT-F: 5’-TAATACGACTCACTATAGGGGTGTCAAGGGACACACTGCTGTTTTAGAGCTAGAAATAG-3’
sgRNA IVT-R:
5’-AAAAGCACCGACTCGGTGCC-3’

### Zygote or two-cell injection and embryo transplantation

Four-week-old BDF1 female mice were super ovulated and mated with BDF1 male mice overnight. Fertilized embryos were collected. For zygote and two-cell injection, the mixture of CGBE mRNA (50 ng/μl) and sgRNA (50 ng/μl) was injected into the cytoplasm of embryos in the droplet of M2 medium containing 5 μg/ml cytochalasin B (CB) using a FemtoJet microinjector (Eppendorf). The injected embryos were cultured in KSOM medium at 37 °C under 5% CO_2_ in air for 24 h (zygote) or 2 h (two cell) and then transferred into oviducts of pseudopregnant ICR females.

### FACS for GOTI

We apply GOTI method to determine genome-wide off-target of CGBEs according to previous study^31^. Breifly, the mixture of CGBE mRNA, sgRNA and Cre mRNA was injected into one blastomere of a two-cell embryo, derived from Ai9 stain male mice mating with 4-week-old wild-type C57BL/6 J female mice. The injected embryos were transferred into oviducts of pseudopregnant ICR females. To isolate mouse embryonic cells, the prepared tissues were cut into small pieces and dissociated enzymatically with 5 mL 0.05% trypsin (Gibco) at 37 °C for 30 min. The digestion was stopped by adding 5 mL of DMEM with 10% FBS. Fetal tissues were then homogenized with 1 mL pipette tips. The cell suspension was centrifuged for 6 min (200 g), and the pellet was resuspended in 2 ml DMEM. Finally, the cell suspension was filtered through a 40 μm cell strainer, and tdtomato^+^ and tdtomato^−^ cells were isolated by FACS. Samples were found to be >95% pure when assessed with a second round of flow cytometry and fluorescence microscopy analysis. Genomic DNA from sorted cells was extracted using the DNeasy Blood and Tissue Kit according to the manufacturer’s instructions. The gating strategy for the separation of tdTomato^+^ and tdTomato^−^ cells was supplied in Supplementary Fig. 2c.

### Target sequencing of endogenous sites

The mCherry and GFP double-positive cells were isolated by FACS at 48 h after transfection. Genomic DNA was extracted by using TIANamp Genomic DNA Kit (TIANGEN) according to the manufacturer’s protocols. Target sites were amplified by nested PCR using site-specific primers (Supplementary Data 2). The PCR reaction was performed for two rounds. Every round was performed at 95 °C for 3 min, 30 cycles at 95 °C for 30 s, 59 °C for 30 s, 72 °C 60 s, and a final extension at 72 °C for 5 min. PCR products were purified using universal DNA purification kit (TIANGEN) according to the manufacturer’s instructions. The amplicons were ligated to adapters and sequencing was performed on the Illumina HiSeq X Ten platforms.

### Whole-genome sequencing (WGS) and RNA-seq

Genomic DNA was extracted using DNeasy blood and tissue kit (Qiagen) according to the manufacturer’s protocols. WGS was performed by Illumina HiSeq X Ten. Total RNA was extracted from mCherry and GFP double-positive cells (~500,000; top 5%) according to the standard protocol for RNA-seq. Sequencing was performed on the Illumina HiSeq X Ten platform. The gating strategy in the identification of GFP^+^ and mCherry^+^ cells RNA off-target effects analysis was supplied in Supplementary Fig. 2b.

### WGS and RNA-seq data analysis

WGS was performed at mean coverages of 50× by Illumina HiSeq X Ten. BWA (v0.7.16) was used to map qualified sequencing reads to the reference genome (mm10). The mapped BAM files were then sorted and marked using Picard tools (v2.25.5). To identify the genome-wide de novo SNVs with high confidence, we conducted single nucleotide variation calling on three algorithms, Mutect2 (v4.2.0.0), Lofreq (v2.1.5), and Strelka (v2.9.10) with default parameters, separately^32–34^. The overlap of three algorithms of SNVs were considered as the true variants.

For RNA-seq data analysis, FastQC (v0.11.3) and Trimmomatic (v0.36)^35^ were used for quality control. Qualified reads were mapped to the reference genome (Ensemble GRCh38) using STAR (v2.7.1)^36^ in 2-pass mode with default parameters. Picard tools (v2.25.5) was then applied to sort and mark duplicates of the mapped BAM files. The refined BAM files were subject to split reads that spanned splice junctions, local realignment, base recalibration, and variant calling with SplitNCigarReads, IndelRealigner, BaseRecalibrator, and HaplotypeCaller tools from GATK (v4.2.0.0)^37^, respectively.

### Target sequencing data analysis

Sequencing data were firstly demultiplexed by Cutadapt (v2.8) and in-house script according to sample barcodes. Target sequences with fewer than 100 reads were discarded to ensure the accuracy of statistics. The demultiplexed reads were then processed by CRISPResso2 for the quantification of mutations, insertions, and deletions at each target site^38^. The on-target editing efficiency was calculated by the number of reads containing only the target mutations divided by the total number of reads. The indel frequency was calculated as the number of reads including indels divided by the total number of reads.

### Sequence motif models

We randomly sampled 80% target sites (1470) and applied a logistic regression model to predict the C-to-G transversion efficiency ranged from 0 to 1. Features were obtained by one-hot-encoding nucleotides per position relative to the targeted C nucleotides within the positions 4–7. The remaining 20% target sites (368) were used as the test set for calculation of R by Pearson’s correlation coefficient.

### CGBE-SMART model

We designed and implemented a deep-learning model, CGBE-SMART, which uses nearby sequences of a target site to predict the substitution frequency of base editing results. The model predicts substitutions from protospacer positions 1–20. Inspired by Google inception networks^39^, we designed a series of networks with different window size for each position. The final output is the weighted average of the results from these networks. The model is trained by minimizing the mean square error (MSE) between observed data and predicted values. Since the model can be trained on different observed data, this could be a powerful and general method for modeling and comparing different base editors.

The whole model is implemented in python based on pytorch. Each nucleotide in the original sequence is first embedded into a vector of length 16. For every nucleotide, we build 9 base models with window size 7, 9, 11 each three. Each base model is coupled with a learned weight towards producing the final outcome. For a base model, the first and second layer contains 256 and 128 neurons using ReLU activation, respectively. The third layer only contains a single neuron which outputs the prediction using Sigmoid activation. Finally, the results of base models are averaged using the learned weights. During training, a dropout rate of 30% is used by default to prevent overfitting.

We consider the editing of each position as a Bernoulli distribution. In order to further output the proportion of all outcomes, we need to further model the dependency between each position. A Markov network is introduced to model such dependency. To simplify this problem, we only consider the relation between adjacent editing positions. Such Markov network is equivalent to a Bayesian network, which is much easier to learn and perform probabilistic inference. The probability of each position being edited can be obtained from the neuronal network model above. The correlation between different editing position is estimated using ${{{{{\rm{c}}}}}}=\frac{{p}_{11}{p}_{00}}{{p}_{01}{p}_{10}}$ from the training set. Here ${p}_{11}$ and ${p}_{00}$ denotes the two positions being edited or not simultaneously, and ${p}_{01}$ and ${p}_{01}$ denotes the two position being edited separately.

The above learning process can be formulated as follows. For a sequence s, the editing positions are denoted as as ${{{{{{\rm{X}}}}}}}_{1},{{{{{{\rm{X}}}}}}}_{2},..{{{{{{\rm{X}}}}}}}_{{{{{{\rm{n}}}}}}}$. The joint probability of the Bayesian network is defined as $p\left({{{{{{\rm{X}}}}}}}_{1},{{{{{{\rm{X}}}}}}}_{2},\ldots ,{{{{{{\rm{X}}}}}}}_{n}\right)=p\left({{{{{{\rm{X}}}}}}}_{1}\right)p\left({X}_{2},|,{{{{{{\rm{X}}}}}}}_{1}\right)\cdots p\left({{{{{{\rm{X}}}}}}}_{n-1},|,{{{{{{\rm{X}}}}}}}_{n}\right)$. The editing efficiency ${{{{{\rm{p}}}}}}\left({{{{{{\rm{X}}}}}}}_{{{{{{\rm{i}}}}}}}=1\right)$ of each position is estimated by the output of the neuronal network ${{{{{\rm{g}}}}}}\left({{{{{\rm{s}}}}}},{{{{{{\rm{X}}}}}}}_{{{{{{\rm{i}}}}}}}\right)$, for ${{{{{\rm{i}}}}}}=1,2,\ldots ,{{{{{\rm{n}}}}}}$. The conditional probability ${{{{{\rm{p}}}}}}\left({{{{{{\rm{X}}}}}}}_{{{{{{\rm{i}}}}}}},|,{{{{{{\rm{X}}}}}}}_{{{{{{\rm{i}}}}}}-1}\right)$ can be learned by preserving the correlation ${{{{{\rm{c}}}}}}$ between position ${{{{{{\rm{X}}}}}}}_{{{{{{\rm{i}}}}}}},{{{{{{\rm{X}}}}}}}_{{{{{{\rm{i}}}}}}-1}$. The proportions of all outcomes can be then inferred from the Bayesian network.

### Training models from exogenous libraries

Datasets are assembled where each gRNA-target pair is matched with a table of observed edited read counts at each position. Reads with indels are discarded. For an experimental replicate, we dropped datapoints with fewer than 100 reads. Data from multiple experimental replicates are then combined by summing read counts for each observed genotype. Since C to G editing is much more difficult, some positions at some target sites observed 0 edited counts. For the convenience of frequency calculation and subsequent analysis, we applied smoothing by adding one count to every edited outcome.

We use the deep conditional autoregressive model from BE-Hive and our CGBE-SMART model to learn the frequency distribution of base editing outcomes. In the original BE-Hive model, both C and G are considered as substrate nucleotides. Herein, we separate reads into reverse and forward directions and transform all reverse reads into forward formats. In this way, only C is considered as the substrate nucleotide. All other hyperparameters accord with the original paper.

Since the proportion of different positions have a big influence on results, splitting the dataset randomly into training and testing sets may not be very appropriate. Herein, we split the dataset into trisection and each time use two for training and one for testing. During training, 10% of the training set is used for validation. At last, the three results are merged to yield the final result. For BE-Hive, we use the default configurations for training. As for benchmarking, we use Pearson correlation and Root Mean Square Error between observed and predicted values for evaluation. The model with the highest performance on the validation set during training process is used in the final benchmarking.

### Comparison with BE-Hive and DeepCBE models

We compared the performance of CGBE-SMART with BE-Hive and DeepCBE from previous studies^26,27^ using the above exogenous libraries. We applied the default configurations for training BE-Hive and DeepCBE models. As for benchmarking, we use Pearson correlation and root mean square error between observed and predicted values for evaluation. The model with the highest performance on the validation set during training process is used in the final benchmarking.

To further compare the three models, we used CBE datasets from BE-Hive and DeepCBE from previous studies. In total, four datasets are included: HEK293T_12kChar_BE4 (BE4), HEK293T_12kChar_BE4-CP1028 (BE4-CP), and HEK293T_12kChar_BE4max_H47ES48A (BE4-max) are from BE-Hive and HT_CBE_Test from DeepCBE. The four datasets contain 7156, 5925, 1785, and 4459 gRNA-target pairs, respectively. We split the datasets into proportions of 6:1:3 for training, validating and testing. The same splitting is used for the training of all three models. Finally, the efficiency for each position in the editing window is inferred and compared.

### Testing models with endogenous data

We use the same model and data processing pipelines on endogenous data as exogenous data. In this section, the model is trained on exogenous data but evaluation is carried out on endogenous data. During the training progress, the dataset is split into a ratio of 4:1 for training and validation. All hyperparameters and training configurations accord with the last section.

The evaluation metrics for testing endogenous data is the average Pearson correlation between observed and predicted frequency of each edited position at the target site. The root mean square error is not applied here due to the heterogeneity of endogenous and exogenous environments. Although a larger absolute error is observed, there is still a strong correlation between predicted and observed values.

### Statistical analysis

R version 4.0.1 (http://www.R-project.org/) was used to conduct all the statistical analyses in this work. All tests conducted were two-sided, and the difference was considered significant at *P* < 0.05. In box-and-whisker plots, the center line indicates the median, the bottom and top lines of the box represent the first quartile and third quartile of the values, respectively. The bottom and top lines represent the minimum and maximum values.

### Reporting summary

Further information on research design is available in the Nature Research Reporting Summary linked to this article.

## Supplementary information

Supplement Information

Description of Additional Supplementary Files

Reporting Summary

Supplementary Data 1

Supplementary Data 2

## Data Availability

Source data are provided with this paper as Source Data files. All the raw sequencing and processed data generated in this study have been deposited in the NCBI Sequence Read Archive (SRA) under accession PRJNA749814 and National Omics Data Encyclopedia (NODE) database under accession code OEP001625. Source data are provided with this paper.

## Source data

Source Data

## Code availability

The website of CGBE-SMART (http://www.sunlab.fun:3838/BE_SMART/) facilitated the practical application of our model. Source code for CGBE-SMART is available on github (https://github.com/tyfei216/BE-SMART).
