# Supplementary material for: Optimization of C-to-G base editors with sequence context preference predictable by machine learning methods
Source: Nat Commun. 2021 Aug 12;12:4902. doi: 10.1038/s41467-021-25217-y (PMC8361092; doi:10.1038/s41467-021-25217-y)
Supplement: Supplementary file 1 — Supplement Information [file 41467_2021_25217_MOESM1_ESM.pdf]

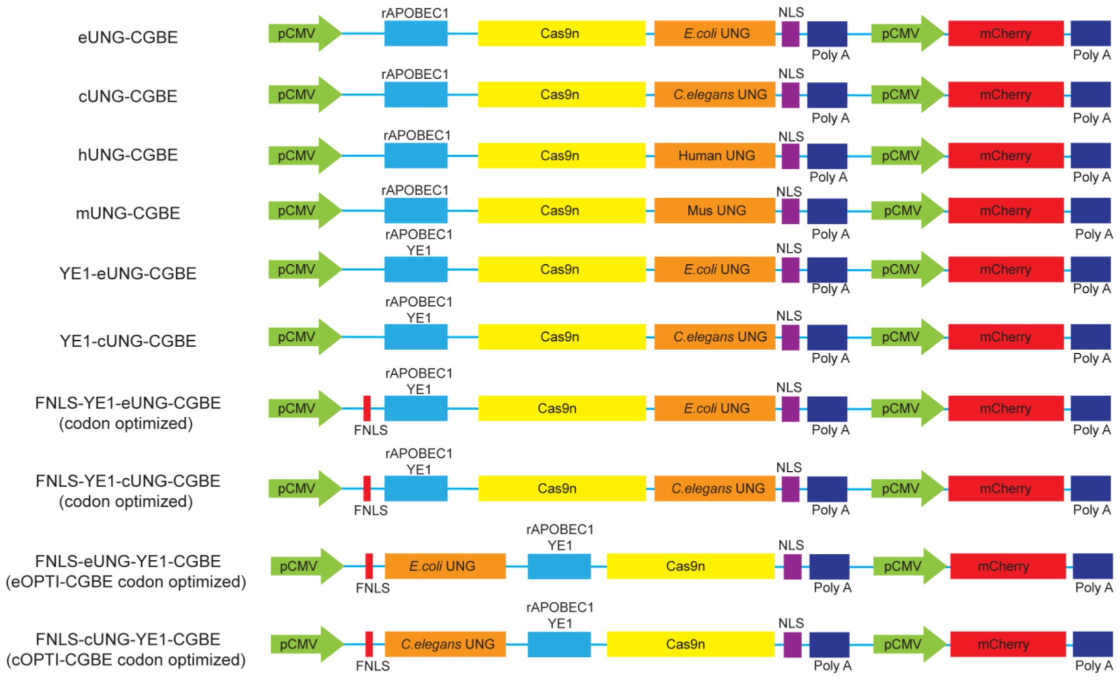

Supplementary Figure 1. Diagrams of engineered CGBEs.

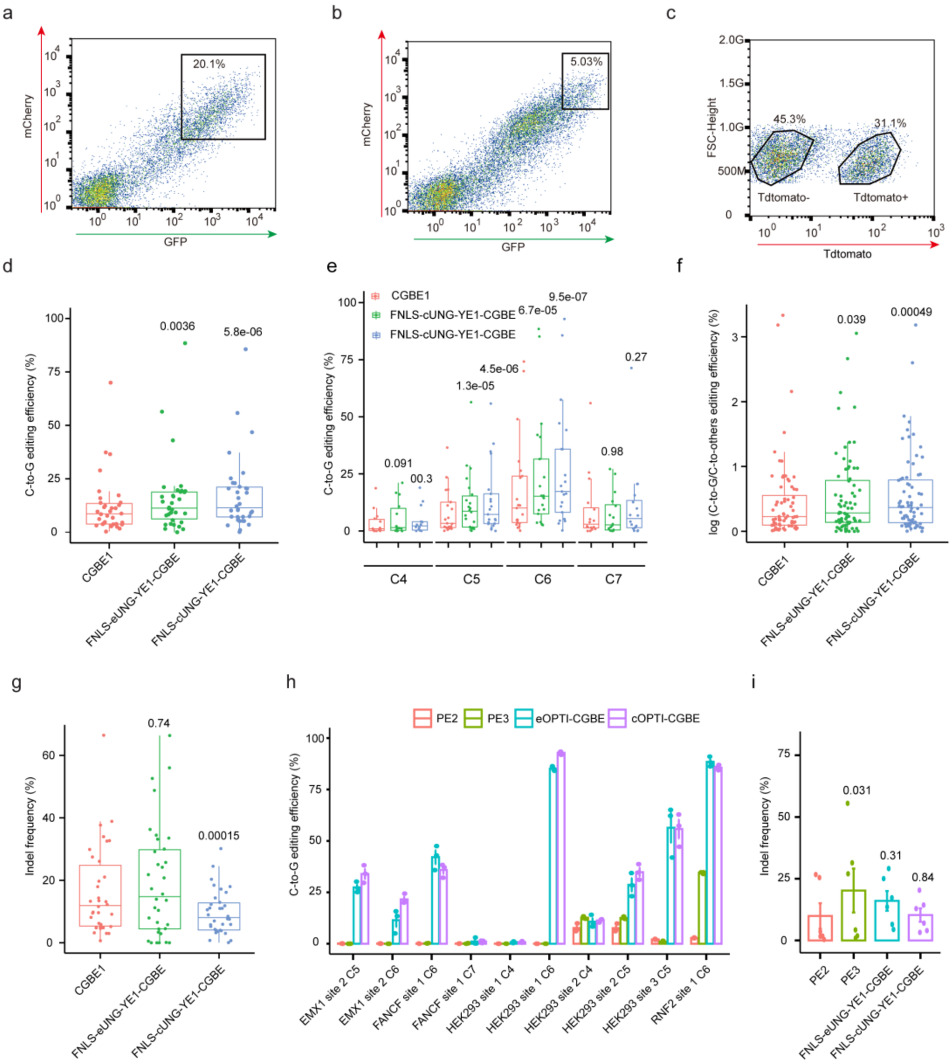

Supplementary Figure 2. Comparison between engineered CGBEs and prime editor. a, The gating strategy in the identification of GFP$^+$ and mCherry$^+$ cells for on-target editing efficiency evaluation. **b**, The gating strategy in the identification of GFP$^+$ and mCherry$^+$ cells RNA off-target effects analysis. **c**, The gating strategy for the separation of tdTomato$^+$ and tdTomato$^-$ cells. **d**, The C-to-G transversion efficiency of R33A and our optimized CGBEs at 34 endogenous target sites in HEK293T cells. **e**, The C-to-G transversion efficiency of different CGBEs at each targeted C within the editing window (where PAM is at positions 21–23) across 34 endogenous target sites in HEK293T cells. **f**, Comparison of C-to-G editing/C-to-others editing ratio among different CGBEs. **g**, Comparison of indel frequencies among different CGBEs. The center line indicates the median, and the bottom and top lines of the box represent the first quartile and third quartile of the values, respectively. Tails extend to the minimum and maximum values. *n* = 3 biological replicates for each site. *P* values above each group indicated the comparison with CGBE1 group. **h**, The C-to-G transversion efficiency of PE2, PE3 and optimized CGBEs. *n* = 3 biological replicates for each site. Data are presented as mean values +/- SEM. **i**, The indel frequencies of PE2, PE3 and optimized CGBEs across the six target sites. *n* = 3 biological replicates for each site. Data are presented as mean values +/- SEM. *P* values above each group

indicated the comparison with PE2 group. All *P* values were calculated by two-sided Wilcoxon rank sum tests.

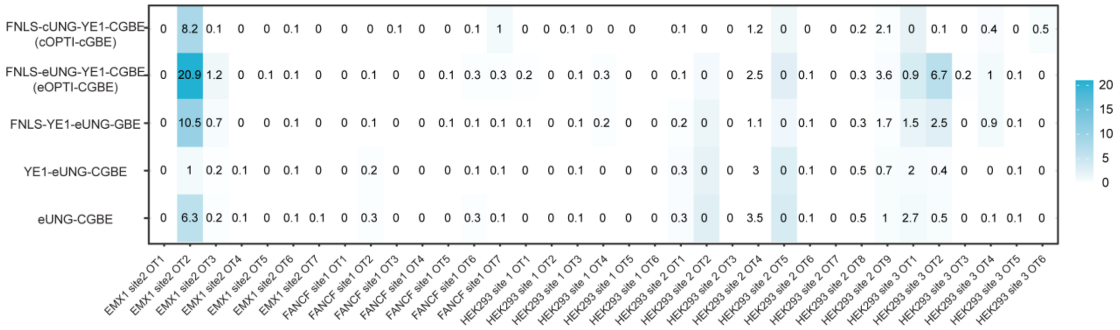

Supplementary Figure 3. The sgRNA-dependent off-target effects of the CGBE variants. n = 3 biological replicates for each site.

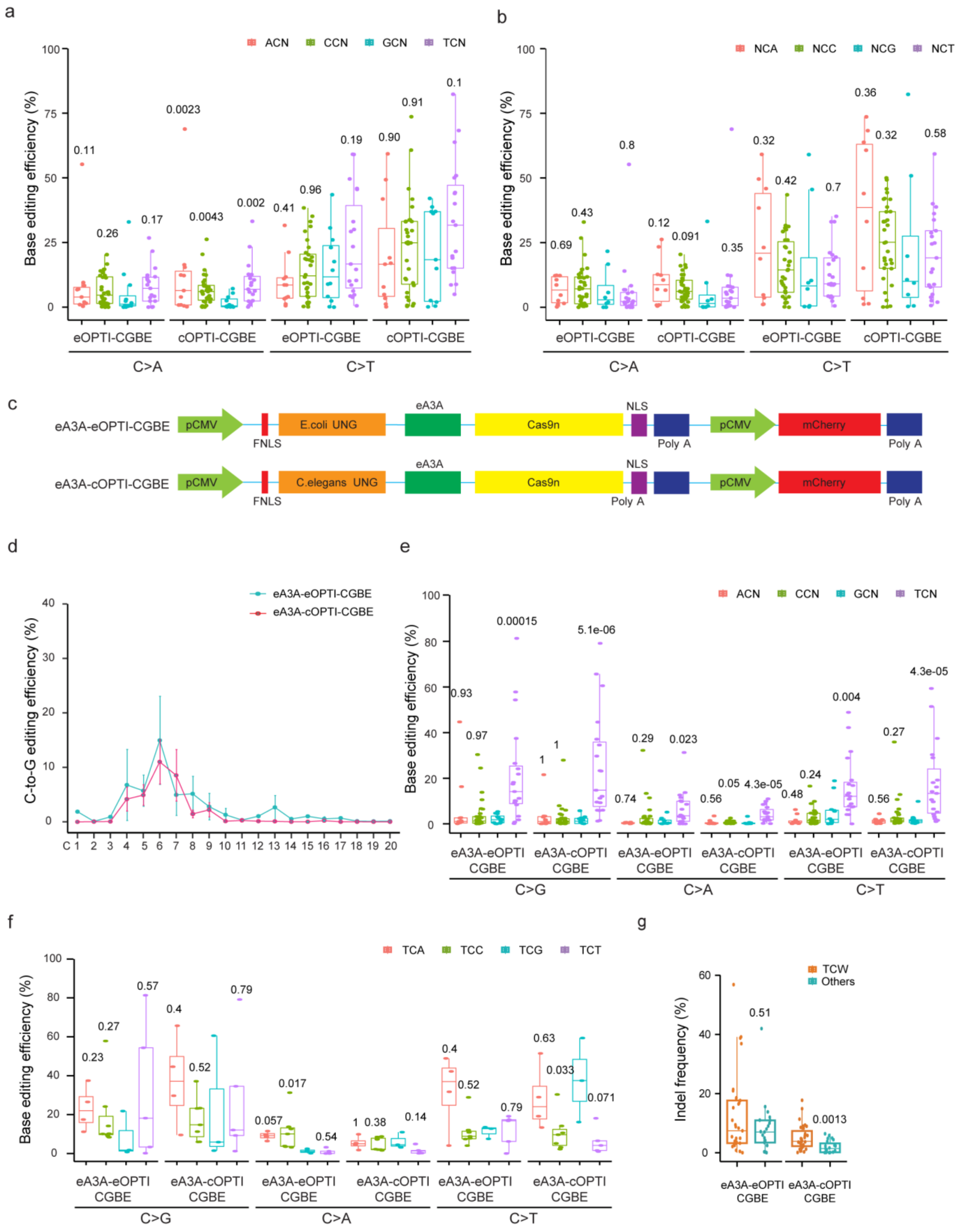

Supplementary Figure 4. The editing outcome of OPTI-CGBEs with eA3A deaminase. a, The base editing efficiency induced by OPTI-CGBEs at targeted Cs bearing different nucleotides 1nt upstream. N = A, T, G or C. *P* values above each group were calculated between the group with “GCN” group. **b,** The base editing efficiency induced by OPTI-CGBEs at targeted Cs bearing different nucleotides 1nt downstream. N = A, T, G or C. The center line indicates the median, and the bottom and top lines of the box represent the first quartile and third quartile of the values,

respectively. Tails extend to the minimum and maximum values. *P* values above each group were calculated between the group with “NCG” group. **c,** Diagram of engineered eA3A-OPTI-CGBE and eA3A-cOPTI-CGBE. **d**, The C-to-G transversion efficiency at each C of protospacers across 34 target sites in eA3A-OPTI-CGBEs. Data are presented as mean values +/- SEM. **e**, The base editing efficiency induced by eA3A-OPTI-CGBEs at targeted Cs bearing different nucleotides 1nt downstream. N = A, T, G or C. *P* values above each group were calculated between the group with “GCN” group. **f**, The base editing efficiency induced by eA3A-OPTI-CGBEs at targeted Cs bearing “TCN” motifs. N = A, T, G or C. *P* values above each group were calculated between the group with “NCG” group. **g**, Indel frequency induced by OPTI-CGBEs with eA3A deaminase at “TCW” and other motif of the 34 original and 20 additional target sites bearing. W = A or T. The center line indicates the median, and the bottom and top lines of the box represent the first quartile and third quartile of the values, respectively. Tails extend to the minimum and maximum values. All *P* values were calculated by two-sided Wilcoxon rank sum tests. *n* = 3 biological replicates for each site.

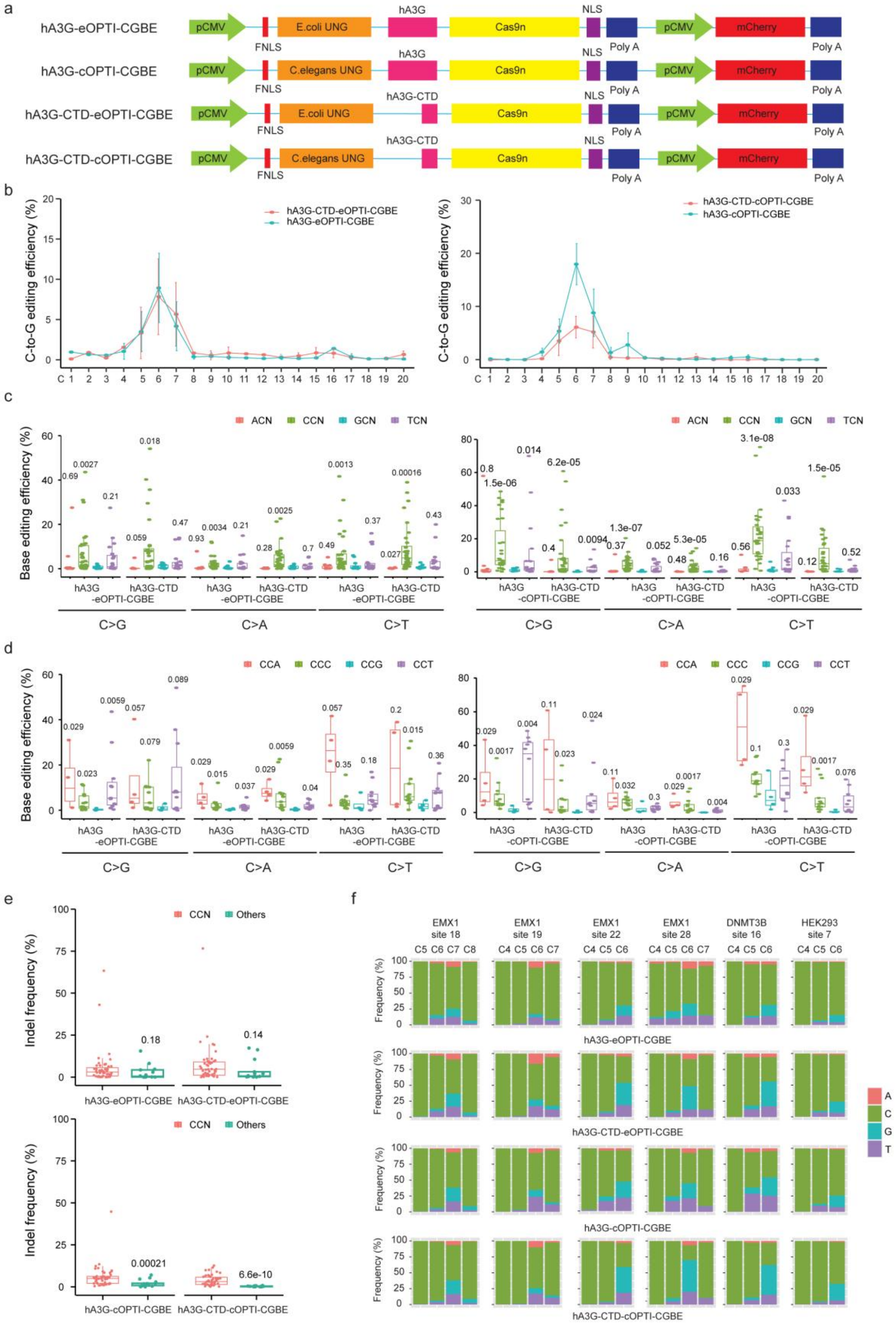

Supplementary Figure 5. The editing outcome of OPTI-CGBEs with hA3G or hA3G-CTD deaminase. a, Diagrams of OPTI-CGBEs with hA3G or hA3G-CTD deaminase. **b**, The C-to-G transversion efficiency at each C of protospacers across 34 target sites in hA3G-OPTI-CGBEs and hA3G-CTD-OPT-CGBEs. Data are presented as mean values +/- SEM. **c**, The base editing efficiency induced by OPTI-CGBEs with

hA3G or hA3G-CTD deaminase of targeted Cs bearing different nucleotides 1nt upstream. N = A, T, G or C. *P* values above each group were calculated between the group with “GCN” group. **d**, The base editing efficiency induced by OPTI-CGBEs with hA3G or hA3G-CTD deaminase of targeted Cs bearing different nucleotides 1nt downstream. N = A, T, G or C. *P* values above each group were calculated between the group with “NCG” group. **e**, Indel frequency of OPTI-CGBEs with hA3G or hA3G-CTD deaminase at the “CCN” or other motif of the 34 original and 26 additional target sites. The center line indicates the median, and the bottom and top lines of the box represent the first quartile and third quartile of the values, respectively. Tails extend to the minimum and maximum values. **f**, Bar plots of representative detailed editing frequency on targeted Cs induced by hA3G or hA3G-CTD deaminase. n = 3 biological replicates for each site. All *P* values were calculated by two-sided Wilcoxon rank sum tests.

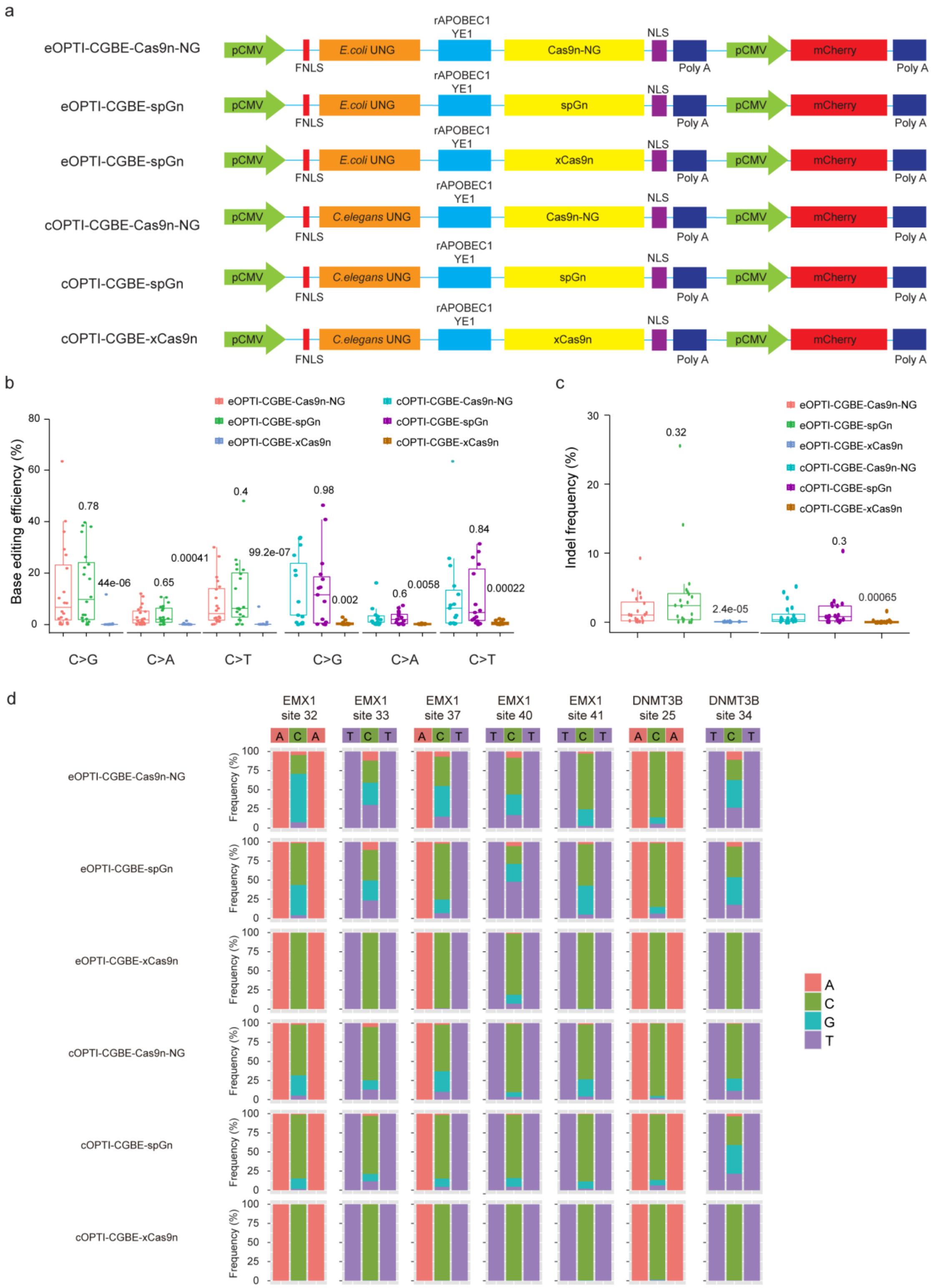

Supplementary Figure 6. The editing outcome of OPTI-CGBE-NGs. a, Diagrams of engineered OPTI-CGBE-NGs. **b**, The base editing efficiency of the 6 OPTI-CGBE-NGs at 20 target sites. N = A, T, G or C. **c**, Indel frequency of 6 OPTI-CGBE-NGs at 20 target sites. The center line indicates the median, and the

bottom and top lines of the box represent the first quartile and third quartile of the values, respectively. Tails extend to the minimum and maximum values. *P* values above each group were calculated between the group with eOPTI-CGBE-Cas9n-NG or cOPTI-CGBE-Cas9n-NG group. **d,** Bar plots of representative detailed editing frequency at targeted Cs induced by 6 OPTI-CGBE-NGs among 20 sites with NG PAM. All *P* values were calculated by two-sided Wilcoxon rank sum tests. $n = 3$ biological replicates for each site.

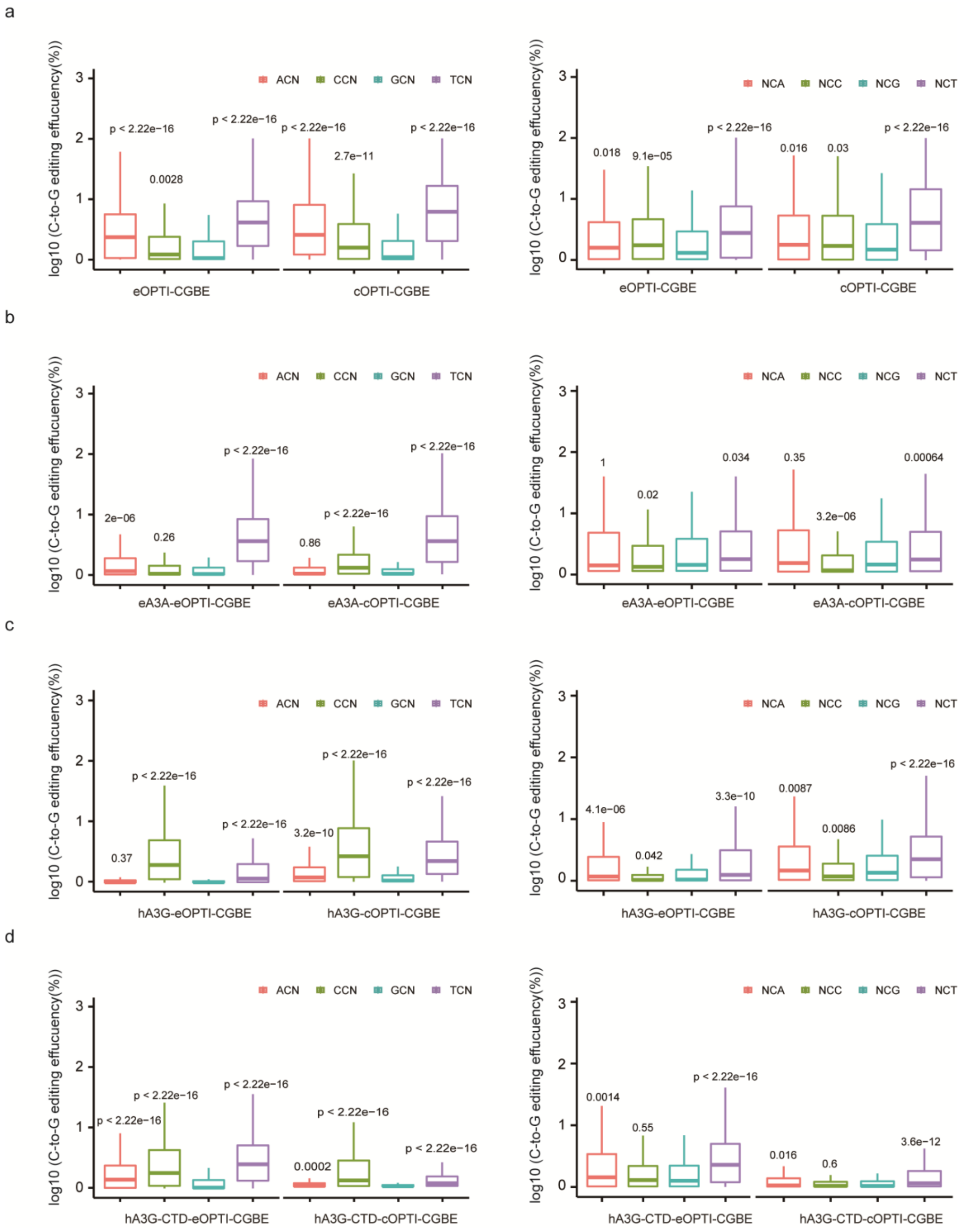

Supplementary Figure 7. Motif analysis of OPTI-CGBEs using paired sgRNA library. a, The C-to-G transversion efficiency induced by OPTI-CGBEs of targeted Cs bearing different nucleotides 1nt upstream or downstream by using paired sgRNA library. N = A, T, G or C. **b,** The C-to-G transversion efficiency induced by OPTI-CGBEs with eA3A deaminase of targeted Cs bearing different nucleotides 1nt upstream or downstream by using paired sgRNA library. **c,** The C-to-G transversion

efficiency induced by OPTI-CGBEs with hA3G deaminase of targeted Cs bearing different nucleotides 1nt upstream or downstream by using paired sgRNA library. **d,** The C-to-G transversion efficiency induced by OPTI-CGBEs with hA3G-CTD deaminase of targeted Cs bearing different nucleotides 1nt upstream or downstream using paired sgRNA library. The center line indicates the median, and the bottom and top lines of the box represent the first quartile and third quartile of the values, respectively. Tails extend to the minimum and maximum values. n = 3 biological replicates for each CGBE. All P values above each group were calculated between the group with "GCN" group or "NCG" group. All P values were calculated by two-sided Wilcoxon rank sum tests.

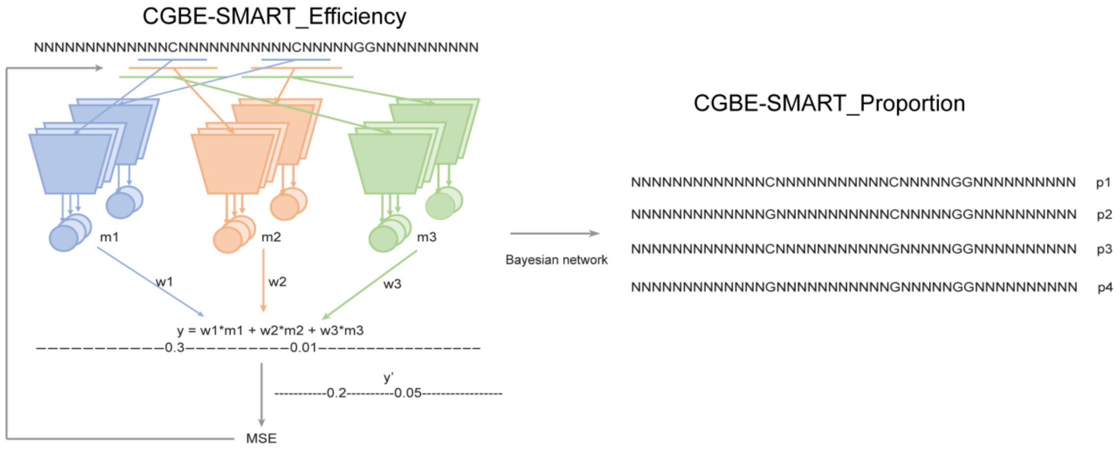

Supplementary Figure 8. Diagram of CGBE-SMART. m = model, w = weight, y = predicted editing efficiency, y' = observed editing efficiency, MSE = mean square error. P = proportion.

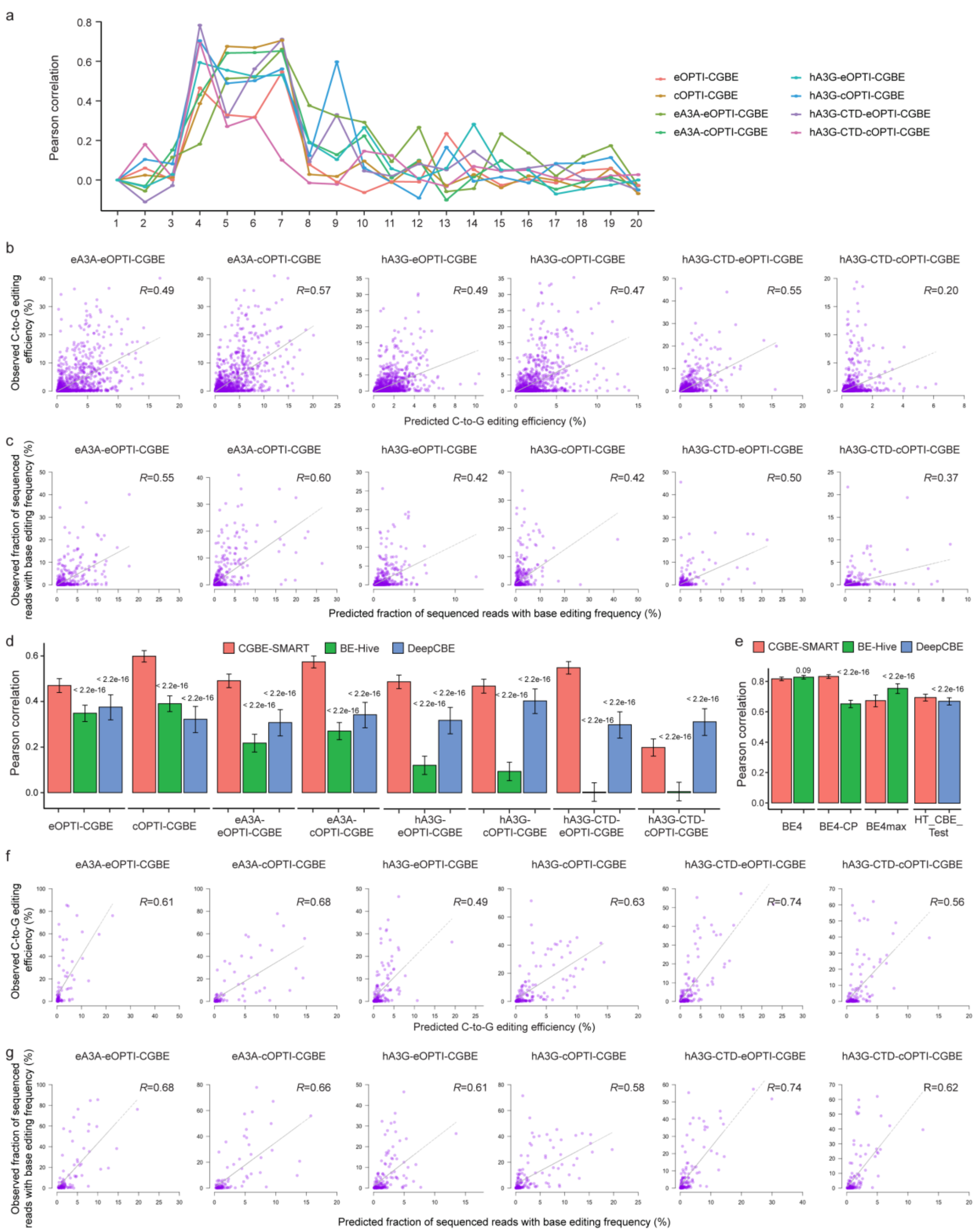

Supplementary Figure 9. Deep learning results of OTPI-CGBEs. a, The pearson correlation coefficients between predicted and observed C-to-G editing efficiency at each target C for OPTI-CGBEs. **b,** Comparison of predicted versus observed base editing efficiency at target sites of OPTI-CGBEs using paired sgRNA library. **c,** Comparison of predicted versus observed fractions of sequenced reads with base editing frequencies of OPTI-CGBEs using paired sgRNA library. **d,** Comparison of

predicted base editing efficiency from CGBE-SMART and BE-Hive or DeepCBE models. **e,** Comparison of predicted base editing efficiency between CGBE-SMART and BE-Hive or DeepCBE models using test datasets from previous studies[10, 11]. Error bars demonstrated 95% confidence intervals. *P* values were calculated by two sided Steiger's Z tests. **f,** Comparison of predicted versus observed base editing efficiency at endogenous target sites of OPTI-CGBEs. **g,** Comparison of predicted versus observed fractions of sequenced reads with base editing frequencies of OPTI-CGBEs at endogenous target sites of OPTI-CGBEs. *R* values demonstrated Pearson correlation coefficients.

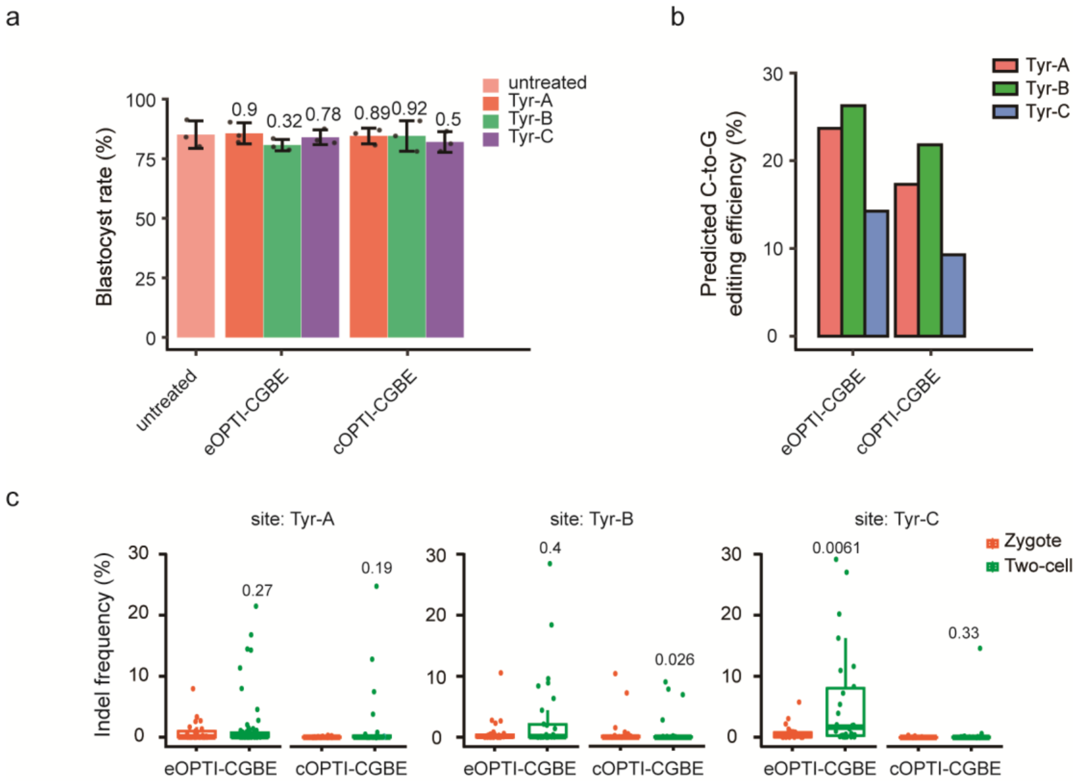

Supplementary Figure 10. Editing outcomes of OTPI-CGBEs in mouse embryos. **a,** Blastocyst rate of mouse embryos injected with eOPTI-CGBE or cOPTI-CGBE mRNA and sgRNA-Tyr. Mean +/- S.E.M.. *n* = 3 biological replicates for each group. **b,** Predicted C-to-G editing efficiency by CGBE-SMART for three target sites. **c,** Indel frequency of eOPTI-CGBE and cOPTI-CGBE at the three target sites on *Tyr* gene. *n* = 30/30/27/27 for Tyr-A, *n* = 28/23/30/21 for Tyr-B and *n* = 21/15/9/15 for Tyr-C, respectively (Ordered: eOPTI-CGBE-Zygote, eOPTI-CGBE-Two-cell, cOPTI-CGBE-Zygote and cOPTI-CGBE-Two-cell). The center line indicates the median, and the bottom and top lines of the box represent the first quartile and third quartile of the values, respectively. Tails extend to the minimum and maximum values. All *P* values were calculated by two-sided Wilcoxon rank sum tests.

Reference

1. Zuo, E.W. et al. GOTI, a method to identify genome-wide off-target effects of genome editing in mouse embryos. *Nat Protoc* **15**, 3009-3029 (2020).
2. Cibulskis, K. et al. Sensitive detection of somatic point mutations in impure and heterogeneous cancer samples. *Nat Biotechnol* **31**, 213-219 (2013).
3. Wilm, A. et al. LoFreq: a sequence-quality aware, ultra-sensitive variant caller for uncovering cell-population heterogeneity from high-throughput sequencing datasets. *Nucleic Acids Res* **40**, 11189-11201 (2012).
4. Saunders, C.T. et al. Strelka: accurate somatic small-variant calling from sequenced tumor-normal sample pairs. *Bioinformatics* **28**, 1811-1817 (2012).
5. Bolger, A.M., Lohse, M. & Usadel, B. Trimmomatic: a flexible trimmer for Illumina sequence data. *Bioinformatics* **30**, 2114-2120 (2014).
6. Dobin, A. et al. STAR: ultrafast universal RNA-seq aligner. *Bioinformatics* **29**, 15-21 (2013).
7. McKenna, A. et al. The Genome Analysis Toolkit: A MapReduce framework for analyzing next-generation DNA sequencing data. *Genome Research* **20**, 1297-1303 (2010).
8. Clement, K. et al. CRISPResso2 provides accurate and rapid genome editing sequence analysis. *Nat Biotechnol* **37**, 224-226 (2019).
9. Szegedy, C. Going deeper with convolutions. *2015 IEEE Conference on Computer Vision and Pattern Recognition (CVPR)* **Boston, MA**, 1-9 (2015).
10. Arbab, M. et al. Determinants of Base Editing Outcomes from Target Library Analysis and Machine Learning. *Cell* **182**, 463-480.e430 (2020).
11. Song, M. et al. Sequence-specific prediction of the efficiencies of adenine and cytosine base editors. *Nature Biotechnology* **38**, 1037-1043 (2020).
