# Supplementary material for: Optimization of C-to-G base editors with sequence context preference predictable by machine learning methods
Source: Nat Commun. 2021 Aug 12;12:4902. doi: 10.1038/s41467-021-25217-y (PMC8361092; doi:10.1038/s41467-021-25217-y)
Supplement: Supplementary file 2 — Description of Additional Supplementary Files [file 41467_2021_25217_MOESM2_ESM.pdf]

Title: Supplementary Data 1:
Description: Amino acid sequence for OPTI-CGBEs

Title: Supplementary Data 2:
Description: gRNA and site-specific primers for PCR reaction.
