# Supplementary material for: Optimization of C-to-G base editors with sequence context preference predictable by machine learning methods
Source: Nat Commun. 2021 Aug 12;12:4902. doi: 10.1038/s41467-021-25217-y (PMC8361092; doi:10.1038/s41467-021-25217-y)
Supplement: Supplementary file 3 — Reporting Summary [file 41467_2021_25217_MOESM3_ESM.pdf]

nature research

Corresponding author(s): Erwei Zuo
Last updated by author(s): Jul 22, 2021

Reporting Summary

Nature Research wishes to improve the reproducibility of the work that we publish. This form provides structure for consistency and transparency in reporting. For further information on Nature Research policies, see our Editorial Policies and the Editorial Policy Checklist.

Statistics

For all statistical analyses, confirm that the following items are present in the figure legend, table legend, main text, or Methods section.

n/a	Confirmed	
☐	☒	The exact sample size (*n*) for each experimental group/condition, given as a discrete number and unit of measurement
☐	☒	A statement on whether measurements were taken from distinct samples or whether the same sample was measured repeatedly
☐	☒	The statistical test(s) used AND whether they are one- or two-sided<br>*Only common tests should be described solely by name; describe more complex techniques in the Methods section.*
☐	☒	A description of all covariates tested
☐	☒	A description of any assumptions or corrections, such as tests of normality and adjustment for multiple comparisons
☐	☒	A full description of the statistical parameters including central tendency (e.g. means) or other basic estimates (e.g. regression coefficient) AND variation (e.g. standard deviation) or associated estimates of uncertainty (e.g. confidence intervals)
☐	☒	For null hypothesis testing, the test statistic (e.g. *F*, *t*, *r*) with confidence intervals, effect sizes, degrees of freedom and *P* value noted<br>*Give P values as exact values whenever suitable.*
☒	☐	For Bayesian analysis, information on the choice of priors and Markov chain Monte Carlo settings
☒	☐	For hierarchical and complex designs, identification of the appropriate level for tests and full reporting of outcomes
☐	☒	Estimates of effect sizes (e.g. Cohen's *d*, Pearson's *r*), indicating how they were calculated

Our web collection on statistics for biologists contains articles on many of the points above.

Software and code

Policy information about availability of computer code

Data collection	Next-generation sequencing data was collected and demultiplexed by Illumina HiSeq X-Ten platform. FACS data was generated using MoFlo XDP (Beckman Coulter) high-speed flow cytometry sorter.
Data analysis	fastqQC (v0.11.3), Trimmomatic (v0.36), BWA (v0.7.16), Picard-tools (v2.25.5), GATK (v4.2.0.0), Mutect2 (v4.2.0.0), Lofreq (v2.1.5), Strelka (v2.9.10), CRISPResso2 (release 20180918), FlowJo X 10.0.7

For manuscripts utilizing custom algorithms or software that are central to the research but not yet described in published literature, software must be made available to editors and reviewers. We strongly encourage code deposition in a community repository (e.g. GitHub). See the Nature Research guidelines for submitting code & software for further information.

Data

Policy information about availability of data

All manuscripts must include a data availability statement. This statement should provide the following information, where applicable:
- Accession codes, unique identifiers, or web links for publicly available datasets
- A list of figures that have associated raw data
- A description of any restrictions on data availability

Source data are provided with this paper as Source Data files. All the raw sequencing and processed data generated in this study have been deposited in the National Omics Data Encyclopedia (NODE) database under accession code OEP001625 [https://www.biosino.org/node/project/detail/OEP001625].

Field-specific reporting

Please select the one below that is the best fit for your research. If you are not sure, read the appropriate sections before making your selection.

- [x] Life sciences
- [ ] Behavioural & social sciences
- [ ] Ecological, evolutionary & environmental sciences

For a reference copy of the document with all sections, see nature.com/documents/nr-reporting-summary-flat.pdf

Life sciences study design

All studies must disclose on these points even when the disclosure is negative.

Sample size	Sample sizes were determined based on literature precedence for genome editing experiments.
Data exclusions	No data was excluded.
Replication	All attempts at replication were successful, n is described or each figure.
Randomization	Due to the small sample, randomization was not relevant for this study. Covariates were controlled for by running controls in parallel whenever applicable.
Blinding	Blinding was not relevant to our study because in general, based on the prior experience of other groups in the field, these types of assays do not require blinding.

Reporting for specific materials, systems and methods

We require information from authors about some types of materials, experimental systems and methods used in many studies. Here, indicate whether each material, system or method listed is relevant to your study. If you are not sure if a list item applies to your research, read the appropriate section before selecting a response.

Materials & experimental systems

n/a	Involved in the study	
x		Antibodies
	x	Eukaryotic cell lines
x		Palaeontology and archaeology
	x	Animals and other organisms
x		Human research participants
x		Clinical data
x		Dual use research of concern

Methods

n/a	Involved in the study	
x		ChIP-seq
	x	Flow cytometry
x		MRI-based neuroimaging

Eukaryotic cell lines

Policy information about cell lines

Cell line source(s)	HEK293T cells were obtained from Cell bank of Shanghai Institute of Biochemistry and Cell Biology, Chinese Academy of Sciences.
Authentication	Cell lines were authenticated with STR profiling by supplier.
Mycoplasma contamination	Cell lines were tested and no contamination of mycoplasma.
Commonly misidentified lines (See ICLAC register)	None of the cell lines used was listed in the database of ICLAC.

Animals and other organisms

Policy information about studies involving animals; ARRIVE guidelines recommended for reporting animal research

Laboratory animals	The following mouse strains were used in the manuscript:<br>C57BL/6J mice: female, 3-4 week-old; Ai9 mice: male , 8-15week-old;ICR mice: females, 8 week-old;<br>BDF1 mice: female,3-4 week-old;BDF1 male: 10-12 week-old.<br>Mice were maintained in a SPF facility under a 12h dark-light cycle.
Wild animals	No wild animals were involved in this study.

Field-collected samples	The study did not involve samples collected from field.
Ethics oversight	The use and care of animals complied with the guideline of the Biomedical Research Ethics Committee of Shanghai Institutes for Biological Science, Chinese Academy of Sciences.

Note that full information on the approval of the study protocol must also be provided in the manuscript.

Flow Cytometry

Plots

Confirm that:

- [x] The axis labels state the marker and fluorochrome used (e.g. CD4-FITC).
- [x] The axis scales are clearly visible. Include numbers along axes only for bottom left plot of group (a 'group' is an analysis of identical markers).
- [x] All plots are contour plots with outliers or pseudocolor plots.
- [x] A numerical value for number of cells or percentage (with statistics) is provided.

Methodology

Sample preparation	HEK293T cells were cultured in Dulbecco's modified Eagle medium (DMEM, Gibco) supplemented with 10% FBS (BI) and 1% penicillin/streptomycin (Gibco) at 37°C in 5% CO2 incubators. The pCMV-CGBE variants-polyA-pCMV-mCherry -polyA and U6-sgRNA-scaffold-pCMV-EGFP-polyA plasmids were co-transfected using polyethyleneimine (PEI, Polyscience) according to the manufacturer's protocols. Forty-eight hours after transfection, cells were washed with PBS and digested with 0.25% trypsin (Gibco). Then cells were filtered with a 40-µm cell strainer. The mCherry and GFP positive cells were sorted by flow cytometer.
Instrument	Cell Sorter (Beckman, MoFlo XDP)
Software	FlowJo X 10.0.7
Cell population abundance	For on-target editing efficiency evaluation, mCherry+ and GFP+ cells (20% of total cells) were selected; For RNA off-target analysis, mCherry+ and GFP+ cells (5% of total cells) were selected. For DNA off-target analysis, Tdtomato+ and Tdtomato- cells were selected.
Gating strategy	For on-target editing efficiency evaluation, positive boundaries were determined by mCherry+ and GFP+ cells. For RNA off-target analysis, positive boundaries were determined by top 5% positive mCherry+ and GFP+ cells (~500,000).For DNA off-target analysis, positive boundaries were determined by Tdtomato+ and Tdtomato- .

- [x] Tick this box to confirm that a figure exemplifying the gating strategy is provided in the Supplementary Information.
